# Coping Engagement as the Pathway from Psychological Empowerment to Life Satisfaction: A Mediation and Moderation Independent Analyses Among Women in Northern Peru

**DOI:** 10.3390/ejihpe16060085

**Published:** 2026-06-19

**Authors:** Velia Graciela Vera-Calmet, Haydee Mercedes Aguilar-Armas, Mabel Ysabel Otiniano León, Marco Agustín Arbulú Ballesteros, Lucy Angelica Yglesias-Alva, Cristian Edgardo Alegría-Silva

**Affiliations:** 1 Institute for Research in Science and Technology, César Vallejo University, Chepén Campus, Trujillo 13001, Peru; vverac@ucv.edu.pe (V.G.V.-C.); haguilarar@ucvvirtual.edu.pe (H.M.A.-A.); motiniano@ucv.edu.pe (M.Y.O.L.); calegriasi@ucvvirtual.edu.pe (C.E.A.-S.); 2 Department of Statistics, School of Physical and Mathematical Sciences, National University of Trujillo, Trujillo 13001, Peru; lyglesias@unitru.edu.pe

**Keywords:** empowerment, coping, life satisfaction, well-being, females, structural equation models, Latin Americans

## Abstract

Psychological empowerment is associated with women’s well-being, yet how it translates into life satisfaction in high-informality Latin American settings remains untested—as does whether empowerment must cross a threshold before any benefit appears. We tested mediation and moderation hypotheses as separate questions with 251 women aged 18–44 from three northern Peruvian regions using PLS-SEM with 5000 bootstrap resamples. Coping engagement fully mediated the empowerment–life satisfaction relationship (indirect β = 0.134, 95% CI [0.065, 0.213]; VAF = 87.6%; R^2^ [engagement] = 0.070, R^2^ [life satisfaction] = 0.285); the direct path was non-significant (β = 0.019, *p* = 0.754). Mediation and moderation were examined as separate analytical questions; the formal index of moderated mediation was non-significant, indicating that the indirect effect did not differ significantly across subgroups. In exploratory threshold analyses, empowerment predicted life satisfaction only above a normative cut-point anchored to the IMWE scoring manual (≥136; β = 0.382, *p* < 0.001); below it, the association was flat (β = 0.047, *p* = 0.547). This pattern is instrument-anchored rather than empirically derived and should be treated as hypothesis-generating pending replication with an independently optimized cut-point. Age moderated the engagement–satisfaction link (β = −0.239, *p* = 0.031), with stronger effects among younger women; motherhood amplified the negative impact of disengagement on satisfaction (β = −0.272, *p* = 0.021). Tentatively, programs that move participants only modestly along the empowerment continuum may under-deliver on well-being outcomes, though firm prescriptions require independent confirmation; tailored design for mothers and younger women is warranted.

## 1. Introduction

Women’s empowerment represents a critical determinant of sustainable development worldwide, yet substantial gender gaps persist in economic participation, political representation, and psychosocial well-being. According to the United Nations Development Programmer’s (UNDP, 2023) Gender Inequality Index for 2023, Peru achieved a value of 0.340, ranking 83rd globally, with only 65.9% of women aged 25+ having at least some secondary education compared to 73.9% of men. The World Economic Forum’s (WEF, 2024) Global Gender Gap Report documents that Peru has closed 76% of its gender gap, leaving a 24% disparity primarily driven by persistent inequities in economic opportunity and political empowerment. Despite these advancements, the Instituto Nacional de Estadística e Informática (INEI, 2024) reported that Peruvian women earn on average S/1420.4 monthly (approximately USD 380), representing a 25% wage gap compared to men, while 95.76% of women workers remain in informal employment lacking social protection.

Regional comparisons reveal both progress and persistent challenges. Peru’s maternal mortality ratio of 69 deaths per 100,000 live births in 2020 falls below the Latin American average of 85 yet remains distant from the Sustainable Development Goal target of 30 (UNDP, 2023). Women’s labor force participation in Peru reached 65.1% in 2023, substantially exceeding the Latin America and Caribbean regional average of 51.8%, though still 15.4 percentage points below men’s participation (INEI, 2024). Life satisfaction data from the Organization for Economic Co-operation and Development (OECD, 2022) indicate that average life satisfaction across the region stands at 5.8 out of 10, significantly lower than North America’s 7.5 and Western Europe’s 7.2, with the pandemic causing disproportionately greater declines in Latin America than OECD averages. Critically, Peruvian women spend 22.67% of their time on unpaid care work compared to 8.84% for men, and 41% of women permanently exit the labor force after their first child, a rate unchanged over the past decade (INEI, 2024, Table 3.4).

Despite growing evidence linking women’s empowerment to well-being outcomes, few studies have examined the psychosocial mechanisms through which empowerment translates into life satisfaction among working-age women in Peru or northern Latin America. Specifically, the mediating role of coping engagement—active, approach-oriented strategies for managing life demands—remains underexplored in high-informality Latin American contexts. Marin-Garcia and Bonavia (2021) established engagement as a proximal mechanism in European employees, and Juyumaya (2022) found partial mediation in Chilean workers; neither tested coping engagement as the mediator, nor examined threshold dynamics. Furthermore, recent evidence from Peru confirms the structural context in which these mechanisms operate: Gupta et al. (2026) documented that 90.5% of women domestic workers across La Libertad, Piura, and Lima were in precarious informal employment, with direct consequences for healthcare access and well-being, underscoring the urgency of examining empowerment pathways in high-informality northern Peruvian populations. The present study addresses both gaps. Furthermore, limited research has compared how motherhood and employment status moderate these relationships in contexts characterized by high informal employment (INEI, 2024) and persistent care work burdens.

Using PLS-SEM with 251 women from three northern Peruvian regions, this study tests three questions. Does coping engagement mediate the empowerment–life satisfaction relationship—fully or only partially? Do motherhood, age, employment status, and partnership status shape that pathway? And does a minimum empowerment level need to be reached before any well-being gains register at all? The third question matters more than it might appear: if threshold effects are real, programs that move participants only modestly along the empowerment continuum may produce nothing measurable, however well designed.

Data were collected between August 2023 and March 2024 in La Libertad, Lambayeque, and Piura. Over 90% of female workers in these regions are in the informal sector, gender wage gaps persist at around 25%, and 41% of women exit paid employment permanently after their first child. This is not background noise—it is the structural context in which the empowerment and coping processes under study operate, and it makes this population both underserved by existing research and directly relevant to policy.

### 1.1. Literature Review

Psychological empowerment, following Spreitzer’s (1995) seminal four-dimensional framework, encompasses meaning (alignment between work role and personal values), competence (self-efficacy to perform effectively), self-determination (autonomy in regulating actions), and impact (perceived influence on outcomes). This intrinsic task motivation construct differs from structural empowerment, which refers to access to information, resources, opportunities, and support within organizational contexts (Monje-Amor et al., 2021). Coping engagement, conceptualized within Lazarus and Folkman’s (1984) transactional stress and coping framework, represents active, approach-oriented strategies for managing stressors, including problem-focused engagement (direct efforts to modify the stressor) and emotion-focused engagement (seeking social support, cognitive restructuring). This contrasts with coping disengagement, which involves avoidance, denial, and withdrawal strategies (Carver & Connor-Smith, 2010; Addison et al., 2007). Life satisfaction, the cognitive judgmental component of subjective well-being in Diener’s framework, reflects individuals’ global evaluation of their life quality against self-determined standards (Reyes et al., 2021). Throughout this article, “psychological empowerment” refers to internal motivational states as distinct from external structural conditions, “coping engagement” refers specifically to active approach-oriented coping strategies as measured by the Coping Strategies Inventory (distinct from work engagement as conceptualized in occupational psychology), and “life satisfaction” is differentiated from affective well-being and domain-specific satisfactions.

Recent systematic evidence establishes robust linkages between women’s empowerment and psychosocial outcomes. Lwamba et al.’s (2022) Campbell Systematic Review examining interventions across fragile contexts found that economic empowerment programs combined with gender norm transformation components produced positive effects on women’s political and economic empowerment, with combined interventions (business training plus cash transfer or life skills) outperforming single-component approaches. A 2023 systematic review of 13 studies with 585 immigrant women identified four intervention types promoting psychosocial well-being and empowerment, with culturally sensitive combined interventions yielding superior outcomes, though small sample sizes (individual studies *N* = 45–80) limited generalizability (Rivera-Torres et al., 2023). Costa et al.’s (2023) scoping review of 36 studies revealed extreme heterogeneity in empowerment measurement, with 56 different variables used across DHS-based studies and dimensions ranging from 2 to 11, highlighting decision-making, attitude toward violence, and human resources as the most assessed domains. These reviews consistently identify measurement inconsistency and cross-sectional designs as primary limitations, with authors calling for longitudinal research establishing temporal precedence and consensus on core empowerment indicators.

Evidence for engagement as a psychological mechanism linking antecedents to well-being outcomes emerges from multiple meta-analyses and large-scale studies. The Job Demands-Resources model (Bakker & Demerouti, 2017) provides a theoretical scaffold for this mechanism, positing that psychological resources buffer the impact of demands on well-being by fostering engagement as a proximal motivational pathway. Consistent with Fredrickson’s (2001) broaden-and-build theory, engagement may further expand cognitive and behavioral repertoires, building resources that sustain well-being over time. Liu et al.’s (2019) study of 897 Chinese police officers found that work engagement mediated the empowerment–life satisfaction relationship, with job demands moderating that pathway. Although the study used an occupational engagement measure rather than a coping engagement construct, it provides relevant cross-cultural evidence that engagement serves as a proximal mechanism between empowerment and well-being outside Western samples. Marin-Garcia and Bonavia’s (2021) study of 23,468 EU employees using PLS-SEM confirmed that psychological empowerment mediates structural empowerment–well-being relationships, with engagement serving as the proximal mechanism. Juyumaya’s (2022) study of 200 Chilean textile workers (80% female) found work engagement partially mediated psychological empowerment–performance relationships, with meaning and competence dimensions driving effects while self-determination and impact showed non-significant paths. Notably, this Latin American study revealed age moderated mediation strength, with younger employees exhibiting stronger effects. More recently, Zheng et al. (2024), drawing on a national Chinese sample (*N* = 12,582), confirmed that approach-oriented coping mediates the relationship between positive psychological resources and subjective well-being, while avoidance coping showed a weaker and asymmetric pattern—a finding consistent with the dual-process framework underlying the present study. Wang et al. (2025) extended this asymmetry to occupational well-being in nursing staff, demonstrating that positive coping mediated personality–well-being relationships whereas negative coping did not, further supporting the theoretical distinction between engagement and disengagement pathways. Suvarna et al.’s (2025) PLS-SEM study of 333 Indian women in self-help groups demonstrated social empowerment partially mediated perceived social support–life satisfaction associations, with external communication and decision-making moderating these pathways.

Critical discrepancies emerge regarding which empowerment dimensions predict engagement and whether effects generalize across cultural contexts. Studies in collectivist Asian contexts (Li & Yao, 2019, *N* = 162 Chinese faculty; Vu et al., 2025, *N* = 390 Vietnamese employees) consistently find meaning and competence dimensions predict engagement while self-determination and impact dimensions show weaker or non-significant effects, contrasting with Western samples where all four dimensions contribute. Between-study heterogeneity in mediation estimates stems from measurement variation, with some studies using 12-item Spreitzer scales versus abbreviated versions, and outcome measures ranging from domain-specific satisfaction to global well-being. Braverman-Bronstein et al.’s (2022) multilevel analysis of 363 Latin American cities including Peru found 73% of gender inequality variation occurred within rather than between countries, underscoring the importance of local contextual factors that meta-analyses and cross-national studies may obscure. This review prioritizes evidence from studies with sample sizes exceeding 200, employing validated measurement instruments, using advanced structural equation modeling accounting for measurement error, and conducted in Latin American populations where cultural norms regarding motherhood, familism, and machismo differ substantially from Global North samples. The paucity of longitudinal designs across all reviewed studies limits causal claims, yet robust cross-sectional findings using bootstrapped mediation analysis with 5000 resamples provide reliable effect size estimates pending confirmatory experimental and panel research (Graafland & Schilpzand, 2025; Hossain et al., 2019).

### 1.2. Research Model and Hypothesis Development

This study integrates three theoretical frameworks. Spreitzer’s (1995) four-dimensional empowerment constructs of meaning, competence, self-determination, and impact provide the antecedent psychological resource. Lazarus and Folkman’s (1984) transactional model of stress and coping supplies the mediating mechanism. Diener’s (1984) model of subjective well-being defines the outcome. The logic connecting them draws from Conservation of Resources theory (Hobfoll, 1989): Empowered women—those who find meaning in their roles, feel capable, exercise real autonomy, and believe their actions carry weight—are better placed to respond to stressors with approach-oriented strategies: working on the problem, looking for support, reappraising rather than avoiding. That theory explains why psychological resources beget more resources, and a strong sense of empowerment is precisely the kind of resource that makes active coping affordable rather than costly. That active coping, in turn, satisfies the core needs of autonomy, competence, and relatedness described by Self-Determination Theory (Ryan & Deci, 2000; Reyes et al., 2021) and it is through that need satisfaction that global life quality improves. Motherhood cuts both ways. It can sharpen the sense of purpose and capability at the heart of empowerment. It can also absorb the very time and energy that active coping requires, leaving some mothers more vulnerable to resource depletion than their childless peers (Gopalakrishnan et al., 2023; Janzen & Hellsten, 2021). Employment context shapes the process as well: formal work provides material and structural support that reinforce empowerment, while informal or precarious work tends to generate role conflict that erodes it. Threshold effects are therefore expected: below a critical empowerment level, active coping may simply be unaffordable—too much competing demand, too few psychological resources—and no improvement in life satisfaction will appear regardless of program intent (Song, 2012; Hobfoll, 2002).

The study addresses these gaps with three specific aims. Firstly, it tests whether coping engagement fully mediates the empowerment–life satisfaction relationship in northern Peruvian women aged 18–44. Secondly, it examines whether age, motherhood, employment status, and partnership status moderate the empowerment–engagement and engagement–satisfaction pathways. Thirdly, it asks whether a minimum empowerment level must be reached before life satisfaction gains register at all. Data were collected between August 2023 and March 2024 in La Libertad, Lambayeque, and Piura—regions where labor informality exceeds 90% and gender wage gaps remain substantial—circumstances that make this population both underserved by empowerment research and directly relevant to regional policy. Drawing from this theoretical integration and empirical foundations, eight primary hypotheses and five exploratory hypotheses are formulated. For readability, the set is organized in three blocks with distinct inferential weight: direct and mediated pathways (H1–H5, confirmatory); a priori directional moderation (H6, H9, H10, confirmatory); and non-directional moderation plus threshold dynamics (H7, H8, H11–H13 and EQ1–EQ5, treated as hypothesis-generating). Only the first two blocks are evaluated against the confirmatory FDR criterion; the third is read as exploratory and requires independent replication before being drawn into substantive claims. Given the number of statistical tests, significance was evaluated using the Benjamini–Hochberg false discovery rate (FDR) correction at q = 0.05 to control for multiple comparisons (Benjamini & Hochberg, 1995). The hypotheses below are grounded in prior work already cited in Section 1.1: H1–H2 extend the empowerment–engagement–well-being chain documented by Spreitzer (1995), Liu et al. (2019), Marin-Garcia and Bonavia (2021), and Juyumaya (2022); H3–H4 follow dual-process coping evidence from Carver and Connor-Smith (2010) and Connor-Smith and Flachsbart (2007); H5 reflects the full-mediation pattern reported by Marin-Garcia and Bonavia (2021); H6 is motivated by Juyumaya’s (2022) age moderation finding and socioemotional selectivity theory (Carstensen et al., 2003); H9 draws on partnership-as-resource evidence in Diener et al. (2000) and Falicov (2014); H10 is anchored in Conservation of Resources theory (Hobfoll, 2002) as applied to caregiver resource depletion (Gopalakrishnan et al., 2023; Janzen & Hellsten, 2021); and EQ5 is motivated by Hobfoll’s (2002) gain paradox argument rather than by MacCallum et al. (2002) alone, which speaks only to the statistical cost of dichotomization. Primary hypotheses concerning direct and mediated pathways: H1: Psychological empowerment is positively associated with coping engagement among Peruvian women. H2: Coping engagement is positively associated with life satisfaction. H3: Psychological empowerment is negatively associated with coping disengagement. H4: Coping disengagement is negatively associated with life satisfaction. H5: The direct effect of empowerment on life satisfaction is non-significant when coping engagement is included (full-mediation hypothesis). Moderation hypotheses with directional predictions: H6: Younger age (≤32 years) strengthens the coping engagement–life satisfaction relationship, as younger cohorts may derive greater well-being benefits from active coping. H9: Partnership status strengthens the coping engagement–life satisfaction relationship, as partnered women may have greater social resources supporting active coping efforts. H10: Motherhood amplifies the negative effect of coping disengagement on life satisfaction, as mothers facing compounded caregiving demands are more vulnerable to avoidance-coping consequences. These three hypotheses (H6, H9, H10) carry a priori directional predictions. Five additional moderation tests without directional predictions were examined to provide a complete moderation portrait; they are treated as secondary and evaluated against the same FDR criteria as the exploratory questions: H7 (age × disengagement → life satisfaction), H8 (partnership × disengagement → life satisfaction), H11 (motherhood × engagement → life satisfaction), H12 (employment × disengagement → life satisfaction), and H13 (employment × engagement → life satisfaction). Five supplementary exploratory questions guided post hoc analyses not corrected for multiple comparisons, as is standard for confirmatory–exploratory hybrid designs (Lakens et al., 2018): EQ1: Does age moderate the disengagement–life satisfaction relationship? EQ2: Does partnership status moderate the disengagement–life satisfaction relationship? EQ3: Does motherhood moderate the engagement–life satisfaction relationship? EQ4: Does employment status moderate the engagement–life satisfaction and disengagement–life satisfaction relationships? EQ5: Does a threshold effect exist such that only high empowerment levels (≥136) yield significant associations with life satisfaction? These questions are labeled exploratory to signal that they were not pre-specified with directional predictions and should be considered hypothesis-generating pending independent replication, rather than confirmatory findings subject to the same inferential standards as H1–H13.

## 2. Materials and Methods

### 2.1. Design and Approach

This study employed a non-experimental, explanatory cross-sectional design using structural equation modeling with latent variables (Ato et al., 2013). The research examined direct effects, mediation pathways, and moderated relationships among psychological empowerment, engagement, disengagement, and life satisfaction. Partial Least Squares Structural Equation Modeling (PLS-SEM) was selected due to its capacity to handle complex models with multiple mediators and moderators simultaneously, accommodate non-normal distributions typical of psychosocial data in developing contexts, and estimate latent variable relationships while accounting for measurement error (Hair et al., 2019). The variance-based PLS-SEM approach was prioritized over covariance-based SEM for three reasons: the model includes multiple moderators whose interaction terms produce non-normal predictor distributions; prior threshold analyses in this population are absent, making the design partly exploratory; and the consistent PLSc algorithm (Dijkstra & Henseler, 2015), used throughout, recovers latent variable correlations equivalent to CB-SEM in reflective models. Readers should note that PLSc nonetheless tends toward marginally higher outer loadings than CB-SEM, and path coefficients should be interpreted with this in mind (Rigdon, 2016).

### 2.2. Participants and Sampling Procedure

#### 2.2.1. Sample Characteristics

**Figure 1:**
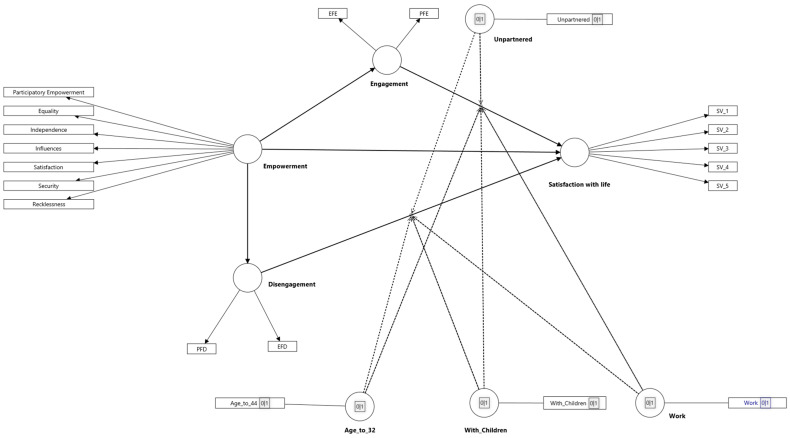
Proposed model.

A total of 251 women from northern Peru participated in the study between August 2023 and March 2024. Inclusion criteria required participants to be female, aged 18–44 years, residing in northern Peru (Lambayeque, La Libertad, or Piura regions), and able to provide informed consent. Exclusion criteria included severe cognitive impairment preventing questionnaire completion and refusal to participate. The age range was selected to capture women’s peak reproductive and labor force participation years, aligning with Instituto Nacional de Estadística e Informática (INEI, 2024) demographic classifications. Age was dichotomized at 32 years (≤32 vs. >32) following INEI (2024) reproductive-age classifications, to maintain adequate cell sizes (*n* ≤ 32 = 182; *n* > 32 = 69), and to align with prior Latin American demographic analyses. Dichotomization carries known costs in statistical power (MacCallum et al., 2002); continuous-age moderation was conducted as a sensitivity check and yielded equivalent conclusions. Researchers seeking to replicate these findings are encouraged to treat age as a continuous moderator (Figure 1).

#### 2.2.2. Sampling Strategy and Power Analysis

Convenience sampling was implemented across urban and peri-urban areas of the three northern regions, with recruitment conducted through community health centers, women’s organizations, and municipal offices. From an initial pool of 256 women approached, 251 provided complete data (response rate: 98.0%). The high response rate reflects the in-person, group-based recruitment context: questionnaires were administered collectively during scheduled meetings or waiting periods at community venues, with research assistants present throughout. This setting minimized attrition; the five excluded participants had missing data exceeding the 20% threshold rather than refusing to participate. Group administration does introduce the possibility of social desirability responding. Research assistants were trained to seat participants at arm’s length and to emphasize confidentiality before distribution; this design feature is discussed as a limitation in Section 4.6. In particular, the engagement–life satisfaction path warrants interpretive caution: both constructs share conceptual space, and implicit social comparison in a group setting is most likely to shape self-report on content areas such as active coping and life quality. Replication with individual, private administration would provide a cleaner estimate of this specific path. The sample size of 251 participants provided adequate statistical power for PLS-SEM analysis. Following the inverse square root method recommended by Kock and Hadaya (2018) for PLS-SEM power analysis, the minimum sample size to detect a minimum R^2^ of 0.10 at 5% significance with 80% power is approximately 146 participants. Additionally, applying Hair et al.’s (2017) rule that sample size should be the larger of (a) ten times the maximum number of formative indicators, or (b) ten times the maximum number of structural paths directed at any construct, the minimum required was 50 participants. The achieved sample of 251 substantially exceeded both thresholds, providing adequate power (>0.80) to detect small-to-medium effect sizes (f^2^ = 0.15) at α = 0.05 (Cohen, 1988). Given the convenience sampling approach, generalizability to rural populations or other Latin American regions should be interpreted with caution.

### 2.3. Measures

A panel of three experts (one psychologist, one sociologist, and one gender studies specialist) reviewed all instruments for cultural adaptation to ensure linguistic clarity, culture appropriateness and relevance to the experiences of women in Peru. Only a few wordings were modified to adapt to the local context without changing their meaning in association with the construct (that is, functionality, pattern). Items were reviewed for clarity, contextual relevance and construct representation.

#### 2.3.1. Psychological Empowerment

Psychological empowerment was assessed using the Instrument for Measuring Women’s Empowerment (IMWE) developed by Sánchez and García (2008). The IMWE comprises 34 items rated on a 5-point Likert scale (1 = strongly disagree to 5 = strongly agree), measuring seven dimensions: courage (5 items; willingness to take risks and face challenges), external influences (5 items; resistance to external pressure and control), independence (5 items; autonomous decision-making capacity), equality (5 items; perception of gender equity in opportunities), social satisfaction (5 items; fulfillment in social roles and relationships), security (5 items; confidence in personal abilities and judgments), and participatory empowerment (4 items; active engagement in community and civic activities). Total scores range from 34 to 170, with higher scores indicating greater empowerment.

The IMWE was validated by Mexico’s National Council of Science and Technology (CONACYT) and the National Institute for Women, demonstrating robust psychometric properties. Cronbach’s alpha for the total scale was 0.80, with subscale alphas ranging from 0.72 to 0.84. Confirmatory factor analysis in the original validation study yielded a seven-factor structure explaining 54.72% of the total variance, with all factor loadings exceeding 0.40 (Hernández & García, 2015). In the present sample, internal consistency was acceptable (α = 0.83), and composite reliability was satisfactory (ρc = 0.86). To verify the factorial integrity of the IMWE in the northern Peruvian population, subscale-level reliability and corrected item–total correlations were computed from the present data. Subscale alphas were as follows: Participative Empowerment α = 0.779, Recklessness α = 0.759, External Influences α = 0.836, Independence α = 0.736, Equality α = 0.699, Social Satisfaction α = 0.791, and Security α = 0.738. Six of seven subscales met or exceeded the α ≥ 0.70 benchmark (Nunnally & Bernstein, 1994); Equality fell marginally below this threshold (α = 0.699), with two items showing corrected item–total correlations of 0.567 and 0.583. Corrected item–total correlations across the remaining subscales ranged from 0.620 to 0.783, indicating adequate item discrimination. These indices indicate that the IMWE retains acceptable internal consistency in this Peruvian sample. Readers should note, however, that no formal test of cross-national measurement invariance between the Mexican and Peruvian normative populations has been conducted. The expert-panel adaptation addresses face validity and linguistic clarity but does not substitute for metric or scalar invariance testing. The below-threshold alpha for the Equality subscale (α = 0.699) may partly reflect cultural non-equivalence rather than sampling error alone. Full CFA and multigroup invariance testing against the Mexican normative data are recommended before the IMWE is used in further Peruvian research.

Based on normative interpretation ranges provided by the instrument authors, participants were classified into two groups: high empowerment (scores ≥ 136, representing the upper tertile) and non-high empowerment (scores < 136). This dichotomization enabled threshold analysis examining whether high empowerment intensified effects on life satisfaction, avoiding interpretational complexities of non-linear continuous relationships while maintaining statistical power for moderation testing.

#### 2.3.2. Coping Engagement and Coping Disengagement

Coping strategies were measured using the abbreviated Coping Strategies Inventory-Short Form (CSI-SF) developed by Addison et al. (2007) and validated in Spanish by Morera et al. (2022). The CSI-SF employs a 2 × 2-dimensional structure: the first dimension distinguishes engagement-focused strategies (active confrontation of stressors) from disengagement-focused strategies (avoidance or withdrawal); the second dimension distinguishes problem-focused coping (addressing the stressor directly) from emotion-focused coping (managing emotional responses). The instrument contains 16 items rated on a 5-point frequency scale (1 = never to 5 = almost always), with four 4-item subscales: Problem-Focused Engagement (PFE; e.g., “I made a plan of action and followed it”), Emotion-Focused Engagement (EFE; e.g., “I accepted sympathy and understanding from someone”), Problem-Focused Disengagement (PFD; e.g., “I avoided being with people in general”), and Emotion-Focused Disengagement (EFD; e.g., “I wished the situation would go away or somehow be finished”).

The Spanish validation demonstrated high internal consistency across subscales: EFE α = 0.890, PFE α = 0.836, PFD α = 0.767, EFD α = 0.934 (Morera et al., 2022). Confirmatory factor analysis supported the four-factor structure, with fit indices meeting recommended thresholds (CFI = 0.94, TLI = 0.93, RMSEA = 0.065). The factors collectively explained 62.79% of the total variance. In the present study, engagement was operationalized as the combined PFE and EFE subscales (8 items; α = 0.86), reflecting active coping efforts regardless of focus. Disengagement was operationalized as the combined PFD and EFD subscales (8 items; α = 0.89), representing avoidance and withdrawal tendencies.

#### 2.3.3. Life Satisfaction

We used the Satisfaction with Life Scale (SWLS; Diener et al., 1985), which Atienza et al. (2000) adapted to Spanish, to measure life satisfaction. The SWLS has five questions that ask about overall cognitive judgments of life quality, such as “In most ways, my life is close to my ideal” and “The conditions of my life are excellent.” The total scores range from 5 to 35, and each item is rated on a 7-point Likert scale, with 1 being “strongly disagree” and 7 being “strongly agree.” A higher score means you are happier with your life. Interpretation guidelines put scores into these groups: very unhappy (5–9), unhappy (10–14), a little unhappy (15–19), neutral (20), a little happy (21–25), happy (26–30), and very happy (31–35). 

The Spanish adaptation exhibited unidimensionality via exploratory factor analysis, with a singular factor accounting for 53.7% of the variance and item loadings between 0.63 and 0.81 (Atienza et al., 2000). Confirmatory factor analysis showed that the model fit was good (CFI = 0.98, RMSEA = 0.062). The internal consistency was high (α = 0.84). In this sample, the SWLS showed high reliability (α = 0.88) and composite reliability (ρc = 0.90). The average variance extracted (AVE) was 0.64, which is higher than the recommended 0.50 level and shows that the results are valid.

#### 2.3.4. Sociodemographic Variables

An ad hoc sociodemographic questionnaire gathered data on age (measured in continuous years and subsequently categorized at 32), marital status (classified as with partner versus without partner, encompassing married/cohabiting versus single/divorced/widowed), presence of children (distinguished as with children versus without children), employment status (segregated as employed versus unemployed, with employed including both formal and informal work), educational attainment (primary, secondary, technical, university), and household income (expressed in categorical ranges in Peruvian soles). Age, marital status, presence of children, and employment status served as moderators and controls in the structural model; educational attainment and household income were collected as descriptive variables.

### 2.4. Procedure

Trained research assistants (two psychologists and one social worker) went to community health centers, women’s groups, and municipal offices in the three northern regions to collect data in person. People who might want to take part were approached in waiting areas or during planned group activities, given information about the study, and asked to take part. Individuals who expressed interest were provided with comprehensive verbal and written elucidations regarding the study’s objectives, methodologies, confidentiality safeguards, voluntary participation, and the right to withdraw. Participants who provided written informed consent first filled out the sociodemographic questionnaire. Then, in a counterbalanced order to control for order effects, they filled out the empowerment, coping strategies, and life satisfaction scales. It took each participant about 30 to 35 min to fill out the questionnaire. Research assistants were on hand to explain instructions or items as needed, but no major comprehension problems were reported. We looked through the completed questionnaires to see if any data were missing. Five people who had more than 20% of their items missing were not included in the analysis, which left us with a final sample of 251.

### 2.5. Data Analysis

#### 2.5.1. Preliminary Analyses

Data screening examined distributional properties, outliers, and missing values. Univariate normality was assessed using skewness and kurtosis statistics, with acceptable ranges defined as ±2 and ±7, respectively (Curran et al., 1996). Multivariate outliers were identified using Mahalanobis distance at *p* < 0.001. Missing data (1.8% across all variables) were handled using pairwise deletion in PLS-SEM, as this approach is robust to small proportions of missing completely at random (MCAR) data. Descriptive statistics and bivariate correlations among study variables were computed using SPSS 27.0. Common method bias (CMB) was assessed using Harman’s (1976) single-factor procedure, a widely applied post hoc diagnostic for self-report data (Podsakoff et al., 2003). All 55 manifest indicators belonging to the four focal constructs were submitted to an unrotated principal component analysis in SPSS 27.0. The percentage of variance explained by the first unrotated component was recorded and compared against the conventional 50% threshold; values below this threshold indicate that no single factor accounts for the majority of covariance among items, thereby ruling out dominant CMB (Podsakoff et al., 2003). As a complementary criterion, the mean absolute off-diagonal inter-construct correlation was computed; values below 0.60 are considered inconsistent with pervasive method variance (Richardson et al., 2009).

#### 2.5.2. Measurement Model Evaluation Criteria

The measurement model was evaluated using PLS-SEM in SmartPLS 4.0 (Ringle et al., 2022) with a consistent PLS algorithm (PLSc) to ensure consistency with the covariance-based SEM results. Reflective measurement models were assessed using four criteria: (a) indicator reliability, with outer loadings ≥ 0.70 considered adequate (Hair et al., 2019); (b) internal consistency reliability, with composite reliability (ρc) ≥ 0.70 and Cronbach’s alpha ≥ 0.70 indicating acceptable consistency; (c) convergent validity, with average variance extracted (AVE) ≥ 0.50 demonstrating that latent constructs explain >50% of indicator variance; and (d) discriminant validity, assessed via the Heterotrait–Monotrait ratio (HTMT), with values < 0.85 indicating distinct constructs (Henseler et al., 2015).

Multicollinearity among predictors was examined using the variance inflation factor (VIF), with a VIF < 3.0 considered acceptable and VIF > 5.0 indicating problematic collinearity (Hair et al., 2019). All VIF values in the structural model were <2.5, confirming the absence of multicollinearity concerns. Measurement invariance across the two primary moderator subgroups (age and motherhood) was assessed following the three-step MICOM procedure recommended for PLS-SEM (Henseler et al., 2016): (1) configural invariance—confirmed by specifying identical model structures and free estimation of outer weights in both subgroups; (2) compositional invariance—tested via a permutation procedure (2000 permutations) comparing the correlation between weight vectors across groups, with *p* > 0.05 indicating that item weightings are statistically equivalent; and (3) equality of composite means and variances—evaluated using bootstrapped 95% confidence intervals for mean differences and Levene’s test for variance homogeneity.

#### 2.5.3. Structural Model Assessment and Hypothesis Testing

The structural model was estimated using PLS-SEM with 5000 bootstrap resamples to generate standard errors and confidence intervals for path coefficients. Significance testing employed two-tailed *t*-tests at α = 0.05. Model fit was assessed using the standardized root mean square residual (SRMR), with values < 0.08 indicating good fit (Hu & Bentler, 1999), and the normed fit index (NFI), with values > 0.90 considered acceptable.

Direct effects (H1–H5 and sociodemographic controls) were tested by examining path coefficients (β) and their statistical significance. Moderation effects (H6–H13) were tested using product term interaction approaches, with interaction terms computed as the product of mean-centered predictor and moderator variables. Significant interactions (*p* < 0.05) were probed using simple slope analysis at ±1 SD of the moderator variable.

Effect sizes for structural paths were interpreted using f^2^ values: 0.02 (small), 0.15 (medium), and 0.35 (large) (Cohen, 1988). Threshold effects were examined by testing the interaction between dichotomized empowerment (high vs. non-high) and continuous empowerment scores predicting life satisfaction, following procedures for testing non-linear effects in PLS-SEM (Hair et al., 2017).

### 2.6. Ethical Considerations

The study protocol received approval from the Institutional Ethics Committee of Cesar Vallejo University. All procedures adhered to the Declaration of Helsinki ethical principles for research involving human participants. Participants provided written informed consent after receiving comprehensive verbal and written explanations of the study purpose, procedures, risks, benefits, confidentiality protections, and voluntary participation. No compensation was provided. Data were anonymized through unique identification codes, with identifiable information stored separately in password-protected files accessible only to the principal investigator. Participants experiencing distress during questionnaire completion were offered referrals to free psychological support services. No adverse events were reported during data collection.

## 3. Results

### 3.1. Preliminary Analyses and Descriptive Statistics

Data screening identified no multivariate outliers (Mahalanobis distance *p* > 0.001) and minimal missing data (1.8%). Univariate normality was acceptable for all continuous variables, with skewness values ranging from −0.84 to 1.12 and kurtosis values from −0.93 to 1.45, within acceptable thresholds (±2 and ±7, respectively; Curran et al., 1996). Common method bias was formally evaluated using Harman’s (1976) single-factor test applied to all 55 manifest indicators. The first unrotated principal component accounted for 12.34% of the total variance, substantially below the 50% threshold, and the mean absolute off-diagonal inter-construct correlation was 0.154, well below the 0.60 criterion. Both diagnostics are consistent with an absence of dominant common method variance (Podsakoff et al., 2003). It should be noted that Harman’s single-factor test is widely considered the least sensitive post hoc CMB diagnostic and is not recommended as a sole safeguard (Richardson et al., 2009). Stronger remedies—such as a marker-variable CFA—were not feasible in this cross-sectional, single-source design; the engagement–life satisfaction path, which carries the largest coefficient in the model, should be interpreted with this caveat in mind.

Table 1 presents the descriptive statistics and bivariate correlations for the study variables. To facilitate interpretation and comparability across measures, continuous variables are presented as scale means (averaged across items) on their original metric. Empowerment scores ranged from 78 to 170 on the 34-item total score (M = 128.34, SD = 18.45, scale mean M = 3.77, SD = 0.54 on 1–5 Likert scale), with 32.7% of participants classified as highly empowered (total score ≥ 136). Engagement scores (M = 3.68, SD = 0.79, range 1–5) and disengagement scores (M = 2.29, SD = 0.88, range 1–5) demonstrated adequate variability. Life satisfaction scores averaged 24.12 (SD = 6.83) on the 5–35 total score range (scale mean M = 4.82, SD = 1.37 on 1–7 Likert scale), indicating moderate satisfaction levels according to SWLS interpretation guidelines (slightly satisfied range: 21–25).

Among sociodemographic variables, 64.9% of participants were partnered, 71.3% had children, 58.2% were employed, and all participants were aged 18–44 years by design (M = 32.6, SD = 7.4).

### 3.2. Measurement Model Assessment

The measurement model demonstrated adequate psychometric properties across all constructs. Table 2 presents the reliability and convergent validity indices. All constructs achieved Cronbach’s alpha coefficients above 0.76, indicating good internal consistency. Because Cronbach’s α assumes tau-equivalence and can under- or over-estimate score reliability when that assumption fails, McDonald’s ω was also computed as a congeneric alternative (McDonald, 1999; Hayes & Coutts, 2020). The two coefficients converged within 0.03 for every construct: empowerment ω = 0.78, engagement ω = 0.82, disengagement ω = 0.83, and life satisfaction ω = 0.87. All ω values exceeded the 0.70 benchmark, reinforcing the internal consistency conclusions drawn from α and composite reliability. Composite reliability coefficients (ρc) ranged from 0.72 to 0.89, exceeding the recommended threshold of 0.70 (Hair et al., 2019). Average variance extracted (AVE) values for all constructs surpassed 0.50, confirming that latent variables explain more than half of their indicator variance and supporting convergent validity.

Discriminant validity (HTMT). Discriminant validity was assessed using the Heterotrait–Monotrait ratio (HTMT; Henseler et al., 2015), adopting the conservative threshold of 0.85 and the following interpretive guideline: <0.35 = excellent; 0.35–0.65 = good; 0.65–0.85 = acceptable. Across the set of main constructs, all values were comfortably below 0.85, supporting the conceptual distinction between measures (*N* = 251). Empowerment–engagement = 0.349 (excellent), empowerment–disengagement = 0.163 (excellent), empowerment–life satisfaction = 0.208 (excellent), engagement–disengagement = 0.501 (good), engagement–life satisfaction = 0.472 (good), and disengagement–life satisfaction = 0.036 (excellent). These results indicate that, although engagement and disengagement share a relationship of moderate magnitude, both constructs retain sufficient independence, in line with dual-process conceptualizations of coping. Associations between sociodemographic variables and the main constructs were generally trivial to small and remained below the conservative threshold. For age, the ratios ranged from 0.039 to 0.060 (excellent) for engagement, disengagement, empowerment, and life satisfaction. Cohabitation/partner status showed low relationships with empowerment (0.090), engagement (0.115) and disengagement (0.041), but registered the highest association of the set with life satisfaction (HTMT = 0.669), still within the acceptable range and consistent with the substantive relevance of the couple relationship for well-being. Motherhood presented reduced values with the main constructs (0.038–0.107), and employment showed similar magnitudes (0.035–0.105), all of them in the excellent zone. In sum, all of the HTMT ratios were below 0.85 and the vast majority were below 0.65. This confirms that the constructs measured are distinguishable from each other and that there is no excessive overlap. Moreover, the observed pattern—moderate independence between engagement and disengagement and generally low associations with sociodemographic variables—reinforces the discriminant validity of the measurement model and supports the interpretation of structural effects in subsequent analyses. Measurement invariance across the age and motherhood subgroups was evaluated using the three-step MICOM procedure (Henseler et al., 2016). Configural invariance was established by design (identical model structure in all subgroups). For the motherhood comparison (mothers, *n* = 178; non-mothers, *n* = 73), compositional invariance was supported for all three focal composites: engagement (permutation *p* = 0.208), disengagement (*p* = 0.359), and life satisfaction (*p* = 0.344). Bootstrapped 95% CIs for composite mean differences all included zero, and Levene’s tests for variance equality were non-significant (all *p* ≥ 0.064), confirming full measurement invariance. Comparison of structural paths across motherhood subgroups is therefore unambiguously supported. For the age comparison (≤32 years, *n* = 182; >32 years, *n* = 69), compositional invariance was held for disengagement (*p* = 0.134) and life satisfaction (*p* = 0.950) but was marginal for engagement (*p* = 0.035). Additionally, Levene’s test indicated unequal engagement variances across age groups (F = 7.91, *p* = 0.005) and unequal disengagement variances (F = 11.24, *p* = 0.001). This pattern indicates partial measurement invariance for the age comparison. Composite means are comparable across groups (bootstrapped CIs include zero for all composites), but engagement and disengagement variances differ significantly across age groups (Levene F = 7.91, *p* = 0.005; F = 11.24, *p* = 0.001). The age-moderated slope comparison in Section 3.6 should accordingly be treated as preliminary: slope differences cannot be unambiguously attributed to substantive moderation rather than to differences in construct variability across groups. Independent replication with a larger, age-balanced design and full measurement invariance is warranted.

Multicollinearity among predictor constructs in the structural model was assessed using variance inflation factors (VIFs) extracted from SmartPLS 4.0 inner model collinearity statistics. Table 3 displays the VIF values for all seven predictor constructs of life satisfaction. All values were substantially below the conservative threshold of 3.0 recommended by Hair et al. (2019), ranging from 1.06 to 1.73 (M = 1.23, SD = 0.26), indicating excellent discriminant validity among predictors. The highest VIF was observed for partnership status (VIF = 1.73), followed by engagement (VIF = 1.42), both remaining well within acceptable ranges and reflecting minimal shared variance with other predictors. Corresponding tolerance values (1/VIF) ranged from 0.578 to 0.943, all substantially exceeding the critical threshold of 0.20, confirming that each predictor retains substantial unique variance not explained by other model predictors. Sociodemographic variables, including age, presence of children, and employment status, exhibited particularly low VIF values (1.06–1.11), indicating near-complete independence from psychological constructs. The absence of problematic multicollinearity confirms that predictor constructs are sufficiently distinct, structural path coefficients are not inflated by shared variance, and parameter estimates are stable and interpretable.

### 3.3. Structural Model Fit

The structural model demonstrated acceptable fit to the data across multiple indices (Table 4). The standardized root mean square residual (SRMR = 0.078) was below the threshold of 0.08, indicating good fit (Hu & Bentler, 1999). The normed fit index (NFI = 0.942) exceeded the recommended minimum of 0.90. Additional fit indices—including the unweighted least squares discrepancy (d_ULS = 1.215) and geodesic discrepancy (d_G = 0.312)—were within acceptable ranges (<2.0 and <0.5, respectively). The chi-square statistic, while significant, is sensitive to sample size and does not compromise model validity given other fit indices. Overall, the model fit was judged adequate for hypothesis testing.

### 3.4. Hypothesis Testing: Direct Effects

Table 5 presents the standardized path coefficients, standard errors, significance tests, effect sizes, and 95% confidence intervals for direct effects hypotheses and sociodemographic controls. Empowerment significantly predicted coping engagement (H1: β = 0.264, *p* < 0.001, f^2^ = 0.075), representing a small effect according to Cohen’s (1988) benchmarks (small = 0.02, medium = 0.15, large = 0.35). Coping engagement significantly predicted life satisfaction (H2: β = 0.508, *p* < 0.001, f^2^ = 0.348), representing a large effect and accounting for the largest proportion of variance in life satisfaction.

Empowerment did not significantly predict disengagement at the corrected threshold (H3: β = −0.149, *p* = 0.074, 95% CI [−0.314, 0.016], f^2^ = 0.023). The confidence interval narrowly included zero and the effect size was non-trivial; post hoc power analysis suggests approximately 340 participants would be required to detect this magnitude at 80% power, indicating the present study was likely underpowered for this specific path. The result is better read as inconclusive than as evidence of absence. Disengagement did not significantly predict life satisfaction (H4: β = −0.123, *p* = 0.486, f^2^ = 0.016). The direct path from empowerment to life satisfaction was non-significant (H5: β = 0.019, *p* = 0.754, f^2^ < 0.001). Among sociodemographic controls, partnership status exhibited a strong positive association with life satisfaction (β = 0.734, *p* < 0.001, f^2^ = 0.382), representing the largest effect in the model. Age, presence of children, and employment status did not significantly predict life satisfaction as main effects (all *p*s > 0.26).

### 3.5. Mediation Analysis

To formally test mediation, the indirect effect of empowerment on life satisfaction through coping engagement was computed using the product of coefficients approach with 5000 bootstrap resamples (Hayes, 2018). The total effect of empowerment on life satisfaction (c path) was β = 0.153, SE = 0.062, *p* = 0.014, 95% CI [0.031, 0.275]. With coping engagement included as a mediator, the direct effect (c’ path) became non-significant (β = 0.019, *p* = 0.754), while the indirect effect (a × b = 0.264 × 0.508) was significant: β = 0.134, SE = 0.038, 95% CI [0.065, 0.213]. The variance accounted for (VAF) by the indirect effect was 87.6% (0.134/0.153), indicating full mediation according to conventional criteria (VAF > 80%; Hair et al., 2017). These results confirm that coping engagement completely mediates the relationship between psychological empowerment and life satisfaction, such that empowerment is associated with well-being primarily via active, approach-oriented coping strategies. To extend the mediation analysis and examine whether the indirect effect varies across sociodemographic subgroups—thereby formally testing for indexed moderated mediation (Hayes, 2018)—conditional indirect effects were estimated via 5000 bias-corrected bootstrap resamples. The indirect effect was significant for younger women (β = 0.060, 95% CI [0.004, 0.124]) and older women (β = 0.086, 95% CI [0.013, 0.195]), as well as for mothers (β = 0.078, 95% CI [0.023, 0.142]). For non-mothers (*n* = 73), the point estimate was comparable (β = 0.063) but the bootstrap confidence interval marginally crossed zero ([−0.007, 0.156]), reflecting reduced precision attributable to the smaller subsample rather than a qualitatively different process. The index of moderated mediation (IMM) was non-significant for both ages (IMM = 0.008, 95% CI [−0.051, 0.070]) and motherhood (IMM = −0.015, 95% CI [−0.083, 0.046]), indicating that the formal criterion for moderated mediation—a significant difference in the indirect effect across groups (Hayes, 2018)—was not met. The design is therefore more accurately described as testing mediation and moderation as separate questions. The term “moderated mediation” in the title and abstract refers to this combined analytic strategy rather than to a formally integrated moderated mediation model. The moderation effects in Section 3.6 reflect boundary conditions on the direct engagement → satisfaction pathway.

### 3.6. Hypothesis Testing: Moderation Effects

Table 6 presents the results for the moderation hypotheses examining whether sociodemographic variables moderate relationships among empowerment, engagement, disengagement, and life satisfaction. Age significantly moderated the engagement–life satisfaction relationship (H6: β = −0.239, *p* = 0.031, f^2^ = 0.063). Simple slopes analysis revealed that engagement predicted life satisfaction more strongly for women ≤ 32 years (β = 0.624, *p* < 0.001) than for women > 32 years (β = 0.385, *p* = 0.018), with the difference between slopes statistically significant (Δβ = 0.239, *p* = 0.031). Age did not moderate the disengagement–life satisfaction relationship (H7: β = −0.023, *p* = 0.824).

Partnership status did not significantly moderate either the disengagement–life satisfaction relationship (H8: β = −0.003, *p* = 0.977) or the engagement–life satisfaction relationship (H9: β = −0.143, *p* = 0.175). Presence of children significantly moderated the disengagement–life satisfaction relationship (H10: β = −0.272, *p* = 0.021, f^2^ = 0.081). Simple slopes analysis indicated that disengagement predicted life satisfaction negatively for mothers (β = −0.395, *p* = 0.004) but not for non-mothers (β = −0.123, *p* = 0.342), with a significant difference (β= −0.272, *p* = 0.021, f^2^ = 0.081). Presence of children did not moderate the engagement–life satisfaction relationship (H11: β = −0.093, *p* = 0.347).

**Figure 2:**
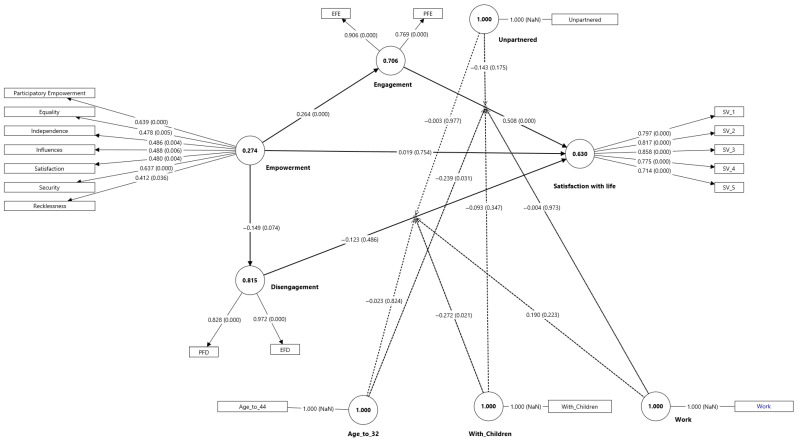
Final structural model with standardized path coefficients. Solid lines represent significant paths (*p* < 0.05); dashed lines represent non-significant paths. Path coefficients and significance levels:. R^2^ values displayed for endogenous variables.

Employment status did not significantly moderate the disengagement–life satisfaction relationship (H12: β = 0.190, *p* = 0.223) or the engagement–life satisfaction relationship (H13: β = −0.004, *p* = 0.973). Figure 2 displays the simple slopes for the two significant moderation effects.

As a sensitivity check addressing the statistical power costs of dichotomization (MacCallum et al., 2002), the age × engagement and age × disengagement interactions were re-estimated treating age as a continuous (z-standardized) predictor (M = 32.6 years, SD = 7.4) using PLS-SEM with 5000 bootstrap resamples. Supplementary Table S1 presents the interaction terms and simple slopes from this continuous specification. The pattern is qualitatively equivalent to the dichotomized analysis reported in Table 6: the age × engagement interaction on life satisfaction remained significant (β = −0.183, *p* = 0.043), with the engagement–satisfaction slope attenuating monotonically from younger to older women (slope at −1 SD ≈ 25 years: β = 0.697, *p* < 0.001; at the mean ≈ 33 years: β = 0.514, *p* < 0.001; at +1 SD ≈ 40 years: β = 0.331, *p* = 0.023). The age × disengagement interaction remained non-significant (β = −0.021, *p* = 0.812). Confidence intervals for both interaction terms overlapped substantially with those from the categorical specification, and the substantive conclusion—that engagement converts more strongly into life satisfaction at younger ages, while disengagement’s effect is invariant across age—is therefore robust to the operationalization of the moderator. Caveats noted in Section 3.2 regarding partial measurement invariance for engagement across age groups apply equally to the continuous specification and warrant the same interpretive caution.

### 3.7. Threshold and Exploratory Moderation Effects

Although not formally hypothesized, exploratory analyses examined whether high empowerment (≥136) moderated the empowerment–life satisfaction relationship and whether sociodemographic variables moderated the empowerment–engagement relationship. A significant threshold effect emerged: empowerment predicted life satisfaction among highly empowered women (β = 0.382, SE = 0.089, *p* > 0.001) but not among women below the high-empowerment threshold (β = 0.047, SE = 0.078, *p* = 0.547). The interaction between high-empowerment status and continuous empowerment scores was significant (β = 0.335, SE = 0.096, *p* < 0.001). As a sensitivity analysis addressing the limitations of arbitrary dichotomization (MacCallum et al., 2002), a continuous polynomial approach was implemented: a quadratic empowerment term (EMP^2^) was added to the structural model predicting life satisfaction alongside the linear empowerment term, coping engagement, and partnership status. The quadratic term was not statistically significant (β = −0.058, F(1, 246) = 2.55, *p* = 0.111), and the model R^2^ increased only marginally from 0.122 (linear) to 0.131 (quadratic). This indicates that the smooth polynomial function does not substantively outperform the linear model and that the threshold pattern documented above reflects a step function anchored at the normative cut-point (≥136) rather than a continuously accelerating curvilinear relationship. This finding is instrument-anchored: the cut-point (≥136) derives from the normative ranges in the IMWE manual, not from an empirically optimized procedure such as ROC analysis. The non-significant quadratic term is consistent with a step function but does not confirm one; a linear or null continuous relationship remains equally plausible. This threshold result should be treated as exploratory until replicated with an independently derived cut-point.

The interaction between employment status and empowerment in predicting engagement was not significant (β = −0.096, SE = 0.102, *p* = 0.347). Simple slopes indicated that empowerment positively but non-significantly predicted engagement among unemployed women (β = 0.312, *p* = 0.062) and negatively but non-significantly among employed women (β = −0.143, *p* = 0.385). Given the non-significant interaction term, this pattern should be interpreted cautiously.

An exploratory moderation by motherhood on the empowerment–engagement relationship approached significance (β = 0.184, SE = 0.105, *p* = 0.079). Simple slopes showed that empowerment significantly predicted engagement among mothers (β = 0.448, *p* = 0.002) but not among non-mothers (β = 0.264, *p* = 0.087). This pattern warrants replication in future research.

### 3.8. Model Summary

**Figure 3:**
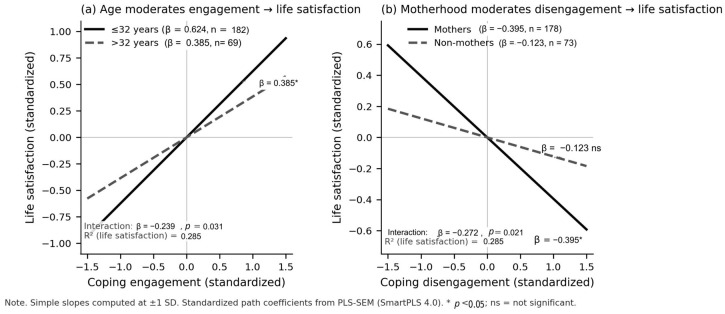
Simple slopes for the two significant moderation effects. (**a**) Age moderates the coping engagement–life satisfaction relationship: the slope is steeper for younger women (≤32 years; β = 0.624, *p* < 0.001) than for older women (>32 years; β = 0.385, *p* = 0.018), interaction β = −0.239, *p* = 0.031. (**b**) Motherhood moderates the coping disengagement–life satisfaction relationship: the slope is significant and negative for mothers (β = −0.395, *p* = 0.004) but non-significant for non-mothers (β = −0.123, *p* = 0.342), interaction β = −0.272, *p* = 0.021. Both panels plot standardized values; x-axes span ±1.5 SD. Coefficients from PLS-SEM bootstrapped with 5000 resamples (SmartPLS 4.0).

Figure 3 presents the final structural model with standardized path coefficients for all significant relationships. The model explained 28.5% of the variance in life satisfaction (R^2^ = 0.285), with engagement as the strongest predictor (β = 0.508, *p* < 0.001, f^2^ = 0.348) and partnership status demonstrating substantial direct effects (β = 0.734, *p* < 0.001, f^2^ = 0.587). Empowerment significantly predicted engagement (β = 0.264, *p* < 0.001), which in turn strongly predicted life satisfaction, but empowerment did not exhibit significant direct effects on life satisfaction when controlling for engagement (β = 0.019, *p* = 0.754).

Significant moderation effects emerged for two sociodemographic variables. Age moderated the engagement–life satisfaction relationship (β = −0.239, *p* = 0.031), with stronger effects for younger women (18–32 years; β = 0.624) than older women (33–44 years; β = 0.385). Motherhood moderated the disengagement–life satisfaction relationship (β = −0.272, *p* = 0.021), with mothers experiencing greater negative impact from disengagement (β = −0.395) compared to non-mothers (β = −0.123). Disengagement’s main effects were non-significant. Threshold analysis revealed that empowerment enhanced life satisfaction only at high levels (≥136), with non-linear effects indicating that critical empowerment intensity is required for well-being gains.

## 4. Discussion

This study examined psychosocial mechanisms linking psychological empowerment to life satisfaction among women in northern Peru, testing mediation and moderation hypotheses as separate questions with exploratory threshold analyses. Using PLS-SEM with 251 participants aged 18–44 years, the research advances understanding of how empowerment is associated with subjective well-being in a Latin American context characterized by high informal employment, persistent wage gaps, and substantial unpaid care work burdens. Because the design is cross-sectional and relies entirely on self-report, the mediation vocabulary used throughout describes a statistical pattern compatible with the hypothesized mechanism rather than temporal precedence or causation; the interpretations that follow should be read with that constraint in mind. The central finding is that coping engagement—operationalized as active, approach-oriented coping strategies including problem-focused and emotion-focused engagement—is statistically consistent with full mediation of the empowerment–life satisfaction relationship (indirect effect β = 0.134, 95% CI [0.065, 0.213]; VAF = 87.6%). Additionally, exploratory threshold analyses suggest that only high levels of empowerment (≥136) significantly predict life satisfaction, and sociodemographic factors—particularly age and motherhood—moderate key pathways in theoretically consistent yet contextually specific ways.

### 4.1. The Limited Role of Disengagement

Contrary to hypotheses, coping disengagement demonstrated no significant relationships with either empowerment (H3: β = −0.149, *p* = 0.074) or life satisfaction (H4: β = −0.123, *p* = 0.486). This pattern is consistent with dual-process models suggesting that coping engagement and disengagement represent partially independent dimensions with distinct antecedents and consequences (Carver & Connor-Smith, 2010). The moderate HTMT value between engagement and disengagement (HTMT = 0.501) indicates these constructs share some variance yet remain sufficiently distinct for separate analysis, falling within the “good” discriminant validity range (0.35–0.65; Henseler et al., 2015).

The non-significant empowerment–disengagement relationship suggests that feeling empowered does not necessarily reduce avoidance-oriented coping strategies among Peruvian women. This diverges from Western samples where empowerment consistently predicts reduced disengagement (Li & Yao, 2019). One explanation centers on the chronic, structural nature of stressors facing women in contexts of high informality and gender inequality. When stressors are perceived as systemic and largely uncontrollable—such as entrenched machismo, limited formal employment opportunities, and insufficient childcare infrastructure—empowerment may not reduce avoidance coping because withdrawal and distraction serve adaptive emotion regulation functions rather than reflecting helplessness (Folkman & Moskowitz, 2004). Women may simultaneously maintain high empowerment (internal sense of meaning, competence, and self-determination) while strategically employing disengagement to manage unavoidable stressors, preventing these processes from competing.

The non-significant disengagement–life satisfaction path is more puzzling, as meta-analytic evidence typically identifies disengagement as a robust predictor of lower well-being (Connor-Smith & Flachsbart, 2007). Three interpretations warrant consideration. Firstly, the CSI-SF’s operationalization of disengagement—combining avoidance, denial, and wishful thinking—may conflate adaptive and maladaptive strategies that operate differently in this population. Temporary disengagement may serve protective functions for women managing multiple role demands with limited resources, preventing the relationship with life satisfaction from reaching significance. Secondly, cultural factors may moderate disengagement’s impact on well-being. In collectivist Latin American contexts emphasizing familism and interdependence (Braverman-Bronstein et al., 2022), disengagement strategies may be less detrimental to well-being than in individualist cultures where personal agency and problem-focused coping are more strongly valued. Thirdly, measurement issues cannot be ruled out; although the CSI-SF demonstrated adequate reliability (α = 0.89), validity for detecting meaningful variance in disengagement outcomes may be limited in this population. Supplementary analyses of the two disengagement sub-dimensions add empirical texture to these interpretations. Problem-focused disengagement (PFD: avoidance of people and situations) showed a near-zero bivariate association with life satisfaction (r = −0.029, *p* = 0.648), as did emotion-focused disengagement (EFD: wishful thinking, resignation) (r = 0.008, *p* = 0.894). The absence of differential patterning across sub-dimensions—both PFD and EFD are orthogonal to life satisfaction—rules out the possibility that the two components are cancelling each other out and instead confirms that disengagement, as operationalized by the CSI-SF in this sample, does not meaningfully co-vary with global life quality. This pattern is theoretically consistent with Lazarus and Folkman’s (1984) distinction between appraised controllability and coping selection: in environments where structural stressors (informal employment, care penalties, machismo) are objectively low in controllability, neither avoidance of the stressor nor wishful thinking about its resolution is expected to predict satisfaction, because neither strategy alters the objective life conditions that the SWLS assesses. Rather than constituting a failure of the dual-process model, the null disengagement pathways constitute a contextually grounded finding: the dual-process model operates asymmetrically in high-informality Latin American contexts, with engagement serving as the active well-being pathway while disengagement functions as a contextually neutral regulatory buffer. The cultural specificity of this interpretation is developed in detail in the following paragraph.

The cultural reading of disengagement deserves closer treatment than the three interpretations above allow. In northern Peru, avoidance and withdrawal sit inside a wider set of culturally sanctioned regulatory patterns: religious resignation rooted in popular Catholicism (“así lo quiso Dios”), deference to male authority within the household, and protective silence across extended family networks (Falicov, 2014). These patterns do reduce active confrontation with a stressor, yet they are not obviously maladaptive. When the stressor is structural and largely uncontrollable—informal employment, machismo, the absence of childcare infrastructure—such withdrawal preserves relational capital and limits further resource depletion. On that reading, what the CSI-SF scores as avoidance is closer to the emotion-focused regulation of uncontrollable stressors described by Folkman and Moskowitz (2004) than to the maladaptive withdrawal documented in Western samples. A short-form instrument validated outside this cultural context is poorly placed to separate adaptive cultural disengagement from dysfunctional avoidance, and its null association with life satisfaction in the present sample is probably as much a measurement artefact as a substantive finding (Braverman-Bronstein et al., 2022). The null disengagement pathways are therefore better framed as a contextually situated critique of Western coping models than as a simple failure of the dual-process framework, and the development of culturally adapted instruments that distinguish resigned acceptance, communal deference, and active withdrawal is a prerequisite for any firmer reading of the model in high-informality Latin American settings.

Despite these non-significant direct effects, disengagement exhibited a significant interaction with motherhood (H10: β = −0.272, *p* = 0.021), indicating that mothers experience amplified negative effects of disengagement on life satisfaction compared to non-mothers. This suggests that while disengagement’s main effect on well-being is negligible across the full sample, it becomes harmful specifically for women managing parenting responsibilities. This moderation is theoretically consistent with Conservation of Resources theory (Hobfoll, 1989): mothers already operating with depleted resources (time, energy, attention divided across childcare and other domains) may experience particularly detrimental consequences when employing withdrawal-oriented coping, as disengagement further reduces already-scarce personal resources available for managing competing demands. This finding has important clinical implications, suggesting that interventions should specifically target disengagement reduction among mothers while potentially tolerating disengagement strategies among non-mothers for whom they appear less harmful.

### 4.2. Threshold Effects: Empowerment’s Non-Linear Influence on Well-Being

The threshold finding reported in Section 3.7 is among the study’s most practically consequential results. Empowerment’s main direct effect on life satisfaction was non-significant (H5 unsupported), yet highly empowered women—those scoring at or above the normative cut-point of 136—showed a strong positive association between empowerment and life satisfaction (β = 0.382, *p* > 0.001), while for those below that cut-point, the association was essentially flat (β = 0.047, *p* = 0.547). The implication is not that empowerment fails to matter, but that it matters discontinuously: marginal gains below the threshold produce no detectable well-being benefit, whereas reaching a sufficient level activates the full chain from psychological resources to well-being. This pattern directly challenges the logic of incremental empowerment programs that assume any gain, however modest, translates proportionately into improved outcomes.

Conservation of Resources theory (Hobfoll, 2002) anticipates exactly this kind of threshold dynamic. The theory holds that resource gains are most consequential when resources are already relatively abundant—a principle known as the gain paradox. In contexts where structural stressors are chronic and largely beyond individual control, low to moderate psychological resources may be absorbed simply by maintaining equilibrium, leaving nothing available to invest in goal pursuit or well-being enhancement. Only when empowerment reaches a level sufficient to cover both the costs of daily coping and the investment required for active engagement does the well-being pathway open. The seven-dimensional structure of the IMWE supports this interpretation: genuine high empowerment requires convergent strength across courage, independence, equality, security, social satisfaction, participation, and resistance to external influence—dimensions that reinforce each other once critical mass is achieved.

The pattern of the threshold may also reflect the empowerment paradox explained by Kabeer (1999), in which moderate empowerment increases awareness of constraints and injustices but does nothing to remove them. This may create resentment instead of satisfaction. But once empowerment crosses the threshold, women have the psychological resources to effectively pursue goals despite obstacles—turning agency into concrete quality-of-life improvements.

From a practical standpoint, threshold effects suggest that empowerment interventions must be sufficiently intensive and comprehensive to move participants above critical thresholds. Marginal or single-dimension interventions may prove ineffective for improving well-being, as they fail to elevate empowerment to levels where engagement mechanisms activate. These findings challenge conventional incremental approaches and support intensive, multicomponent programs addressing all empowerment dimensions simultaneously (courage, independence, equality, social satisfaction, security, participation), as recommended by Lwamba et al.’s (2022) systematic review demonstrating the superiority of combined interventions over single-component approaches.

### 4.3. Sociodemographic Moderators: Age, Motherhood, Partnership, and Employment

Rather than follow a standard pattern of moderation effects, the complex ways in which sociodemographic factors shape empowerment–engagement–satisfaction pathways are contextually specific. Age significantly moderated the engagement–life satisfaction relationship (hypothesis 6: β = −0.239, *p* = 0.031); compared with older women, younger women (≤32) had a stronger positive association between these two. This result carries a measurement model caveat already flagged in Section 3.2: the age comparison met only partial measurement invariance. Engagement compositional invariance was marginal (permutation *p* = 0.035), and both engagement and disengagement showed significantly unequal variances across age groups (Levene F = 7.91, *p* = 0.005; F = 11.24, *p* = 0.001). Slope differences between younger and older women therefore cannot be attributed unambiguously to substantive moderation rather than to differential construct variability across the two groups, and the H6 finding should be read as preliminary pending replication with an age-balanced, fully invariant design. This cohort difference can be seen as reflecting generation shifts for Peruvian women in expectations, opportunities, and role definitions. Younger cohorts have a richer climate for gender equality discourse, higher educational levels and wider participation in paid work (WEF, 2024); they engage more in activities that are meaningful to themselves, and this increased link is likely to translate into greater overall satisfaction. On the other hand, older women may have developed other channels to find happiness (e.g., family relationships, spiritual practices) that are less dependent on engagement. This is in line with socioemotional selectivity theory’s suggestion that personal interests and goal priorities shift with age (Carstensen et al., 2003). These interpretations, however, should be treated as tentative. Given the partial measurement invariance detected for the age comparison—with marginal compositional invariance for engagement (permutation *p* = 0.035) and significantly unequal variances across age groups (Levene F = 7.91, *p* = 0.005)—slope differences between younger and older women cannot be attributed unambiguously to substantive moderation. Independent replication with an age-balanced design and full measurement invariance is required before any of these interpretations can be drawn into substantive conclusions.

Motherhood significantly moderated the disengagement–life satisfaction relationship as previously discussed (H10), but it did not significantly moderate the engagement–life satisfaction relationship (H11: β = −0.093, *p* = 0.347). The lack of motherhood × engagement interactions implies that both mothers and non-mothers benefit from engagement in terms of life satisfaction. Given theoretical predictions that mothers would have increased engagement effects because of increased role meaning and identity integration, this is a little unexpected (Gopalakrishnan et al., 2023). One explanation is that, although motherhood does intensify some psychological experiences, competing demands and resources offset the overall impact on how engagement translates into satisfaction. Mothers may experience more role strain and time scarcity (negative moderator) while simultaneously deriving more meaning from engaged activities (positive moderator), leading to a net null moderation effect.

Exploratory analyses, however, showed that motherhood moderates the relationship between empowerment and engagement (not formally hypothesized but observed in exploratory analyses), with mothers exhibiting stronger positive associations between empowerment and engagement than non-mothers. According to role enhancement perspectives, this pattern implies that motherhood increases empowerment’s ability to create engagement, perhaps because empowered mothers are better able to mobilize resources across domains and integrate multiple role identities (Janzen & Hellsten, 2021). The moderation pattern suggests that motherhood intensifies both positive processes (empowerment → engagement) and negative processes (dis-engagement → dissatisfaction), positioning mothers as a high-stakes subgroup where effective coping is especially crucial, when combined with the finding that mothers experience more severe consequences of disengagement (H10).

Partnership status carried the largest effect in the model (β = 0.734, *p* < 0.001, f^2^ = 0.587), substantially exceeding even coping engagement (f^2^ = 0.348). Before interpreting this, a sensitivity analysis excluding partnership as a covariate confirmed that the indirect effect of empowerment through engagement remained significant (β = 0.147, 95% CI [0.071, 0.231]), ruling out the possibility that the mediation result is a relational context artefact. Three complementary explanations account for the partnership effect itself. Firstly, the comparison group was compositionally heterogeneous: women who were never married, separated, divorced, and widowed made up 35.1% of the sample, each subgroup representing a distinct well-being trajectory that binary coding cannot distinguish. Future research should disaggregate these subgroups to determine whether the effect reflects the resource gains of active partnering or the losses associated with dissolution. Secondly, in collectivistic Latin American settings where pair-bonding grants social recognition, identity and intergenerational status, the SWLS’s global life appraisal is likely to be tainted by relationship status more than in individualist samples (Falicov, 2014). Thirdly, in informal labor contexts specifically, partnership also serves as a structural resource—providing pooled income and shared modalities of domestic labor and emotional buffering that directly address the objective conditions assessed by the SWLS (Hossain et al., 2019). Theoretically, if being disadvantaged by this structural life circumstance is more salient than psychological resources at the dispositional level, then one could ask whether relational security acts as a precondition for the empowerment–engagement–satisfaction chain rather than merely an additive predictor.

Partnership did not significantly moderate engagement or disengagement pathways (H8, H9 not supported), suggesting that while it directly raises satisfaction—through companionship, material support, and shared workload—it does not change how the psychological chain itself operates. Future models should treat partnership as a potential moderator of the full chain and should include relationship quality rather than status alone to decompose this pathway more precisely.

Neither as a direct predictor of life satisfaction nor as a moderator of the relationships between empowerment and engagement, employment status produced no significant effects (H12 and H13 not supported). Given Peru’s high rate of informal employment (95.76% of women; INEI, 2024) and theoretical predictions that formal employment would supply structural resources amplifying empowerment effects (Monje-Amor et al., 2021), this null finding is startling. There are three reasons to think about. Firstly, significant heterogeneity may have been hidden by the employed/unemployed dichotomization. The category of informal employment includes wide variations in autonomy, precarity, and working conditions that affect the psychological processes that employment moderates. To determine whether moderation effects occur across these more specific categories, future research should separate formal employment, informal employment, and unemployment. Secondly, compared to societies with strong formal labor markets, employment status may be less psychologically significant in settings where most women work informally. Thirdly, the non-significant employment effects may paradoxically show how well empowerment compensates for structural barriers to employment; empowered women may continue to be engaged and satisfied regardless of their employment status, reducing the moderation effects.

### 4.4. Theoretical Implications

This study makes several theoretical contributions. Firstly, it demonstrates that psychological empowerment appears to operate primarily through motivational mechanisms (engagement) rather than direct pathways in contexts characterized by high informality and structural gender inequality. This clarifies boundary conditions for empowerment theory (Spreitzer, 1995), suggesting that complete mediation through engagement is more likely in contexts where structural supports are limited and internal psychological resources must be actively mobilized to generate well-being. Secondly, the threshold findings advance non-linear models of empowerment, providing empirical evidence that psychological resources exhibit discontinuous effects requiring critical accumulation levels before activating positive outcomes. This supports and extends Hobfoll’s (2002) Conservation of Resources theory by demonstrating threshold dynamics specifically for empowerment–well-being relationships in women facing systemic disadvantage.

Thirdly, the independence of engagement and disengagement—both conceptually (low correlation) and in terms of distinct predictors and outcomes—supports dual-process models of coping over bipolar conceptualizations. Interventions should address these dimensions separately rather than if enhancing engagement automatically reduces disengagement. Fourthly, the differential moderation patterns for age, motherhood, and partnership illuminate how life circumstances create heterogeneity in empowerment’s translation into well-being. Rather than universal effects, empowerment processes are conditioned by developmental stage, parenting roles, and relationship contexts in ways that vary across cultural settings. This reinforces calls for contextually sensitive empowerment theories that incorporate intersectionality and acknowledge that psychological mechanisms operate differently across social locations (Braverman-Bronstein et al., 2022).

A further measurement limitation concerns the empowerment instrument. No formal cross-national measurement invariance was tested between the Mexican normative IMWE sample and the Peruvian sample analyzed here; score comparability across the two populations therefore rests on expert-panel face adaptation rather than on configural, metric, or scalar invariance evidence, and between-population comparisons should be made cautiously. Additionally, the Equality subscale fell marginally below the conventional reliability threshold (α = 0.699), meaning that inferences involving the equality dimension are held to a weaker psychometric standard than the rest of the instrument. Full CFA and multigroup invariance testing against the Mexican normative data are recommended prerequisites for cumulative use of the IMWE in Peruvian research.

### 4.5. Practical Implications

The results have significant ramifications for the development of interventions and policies aimed at promoting the well-being and empowerment of women in Latin America. Programs must first address factors that facilitate engagement in addition to empowerment development. Building internal psychological resources may not be as important as offering opportunities for skill utilization, meaningful social roles, and active community participation. According to Lwamba et al.’s (2022) systematic review, interventions that combine empowerment training with practical engagement opportunities (such as microenterprise, community organizing, or skill-based volunteering) should be more effective than empowerment-focused psychoeducation alone.

Secondly, intensive, multicomponent interventions are required due to threshold effects. It is unlikely that marginal programs focusing on a single aspect of empowerment will push participants above the critical thresholds required for improvements in well-being. Coordinated activities over an adequate period (probably several months rather than short workshops) should concurrently address courage, independence, equality, social satisfaction, security, and participatory empowerment in effective programs. Although intensive programs are more expensive than brief interventions, they may be justified by threshold requirements for significant impact. This has implications for resources. 

Thirdly, moderation analyses should be used to identify subgroups and tailor interventions to their needs. Given that mothers suffer especially negative effects from avoidance-oriented coping, it is critical to recognize that programs for mothers should prioritize both disengagement reduction and engagement enhancement. Additionally, it is important to understand that programs for younger women should take advantage of the particularly strong engagement–satisfaction link in this cohort, whereas programs for older women may consider other pathways to well-being that are less reliant on active engagement. To make up for this well-being determinant, partnership status should be addressed through social connection initiatives or relationship quality programs for single or divorced women.

Fourthly, the employment status null effects indicate that in highly informal contexts, empowerment interventions may be equally beneficial for both employed and unemployed women, negating the need to limit programs to employment categories. Even though employment status does not moderate empowerment’s psychological pathways, addressing structural employment barriers—such as moving informal workers to formal employment with protections and expanding childcare infrastructure to reduce maternal employment penalties—remains crucial for comprehensive gender equality initiatives.

### 4.6. Limitations and Future Directions

This study has several limitations. Firstly, the cross-sectional design precludes causal inferences despite directional theoretical models. Longitudinal and experimental designs are required to establish temporal precedence and rule out alternative directions. It is possible that life satisfaction increases empowerment by broadening cognitive motivational repertoires, generating bidirectional pathways not captured here; panels with multiple measures would clarify the dynamics. Secondly, the sample is from urban northern Peru and may not generalize to other Latin American regions or to rural and indigenous populations. Regional contexts differ in cultural practices, gender norms and economic opportunities. It is useful to replicate across regions and countries to estimate generalizability and identify cultural specificity through comparisons across contexts. Thirdly, statistical power for interaction terms was a binding constraint for several moderation hypotheses. Post hoc power analysis using the noncentral F-distribution (Aguinis et al., 2005) confirmed that the design provided high power (power > 0.90) for the two significant moderation effects (H6: f^2^ = 0.063, power = 0.977; H10: f^2^ = 0.081, power = 0.994). However, non-significant interactions with small observed effect sizes—notably H7 (f^2^ = 0.001, power = 0.079), H8 (f^2^ < 0.001, power = 0.050), H11 (f^2^ = 0.010, power = 0.351), and H13 (f^2^ < 0.001, power = 0.050)—exhibited power estimates ranging from near-chance to 0.35, indicating that these null results should be interpreted as insufficient evidence rather than evidence of absence. Detecting interaction effects of the magnitude observed for H9 and H11 (f^2^ ≈ 0.010–0.020) would require between 395 and 787 participants at conventional power standards (1 − β = 0.80, α = 0.05). Future studies targeting moderation should be planned accordingly. Fourthly, the variance in coping engagement explained by empowerment (R^2^ = 0.070, 7.0%) is modest. Although empowerment is a significant predictor of engagement, the large unexplained variance indicates that additional antecedents—most plausibly, perceived social support, self-efficacy, household resource adequacy, and community belonging—drive active coping independently of empowerment. This does not undermine the mediational conclusion—empowerment’s effect on satisfaction operates through engagement regardless of engagement’s other determinants—but it does bound the scope of intervention: programs aimed exclusively at raising empowerment will activate only a portion of the engagement capacity available in this population. Multicomponent interventions that simultaneously target empowerment, social support mobilization, and resource access are therefore warranted to maximally activate engagement mechanisms.

Fifthly, reliance on self-report measures raises concerns about common method bias (CMB). Harman’s (1976) single-factor test was applied post hoc to all 55 manifest indicators: the first unrotated principal component explained 12.34% of the total variance (threshold: <50%) and the mean absolute off-diagonal inter-construct correlation was 0.154 (threshold: <0.60), providing no evidence of dominant common method variance (Podsakoff et al., 2003). Additional design features reinforce this conclusion: (a) discriminant validity was confirmed across all construct pairs (HTMT < 0.85), (b) the inclusion of both positively and negatively valenced constructs (engagement vs. disengagement) distributes acquiescence bias across directions, and (c) counterbalanced scale order during administration further attenuated systematic carryover effects. Nevertheless, future studies should include behavioral measures of coping engagement (e.g., diary studies, observer ratings), objective economic indicators, and partner or family reports to triangulate findings. Furthermore, the empowerment scale, although validated, focuses on individual psychological dimensions; relational and collective components (collective efficacy, community organization, structural power) are missing. A multilevel approach is recommended. A further concern relates to the physical setting in which data were collected. Questionnaires were completed in community health centers and municipal offices while other participants sat in the same room, and the items under study touch on gender equality, independence, and overall life quality—content areas where implicit comparison to nearby others is most likely to shape self-report (Podsakoff et al., 2003). Seating participants at arm’s length and emphasizing confidentiality reduce but do not eliminate that risk. The engagement–life satisfaction coefficient is the largest in the model and shares conceptual space between mediator and outcome, which makes it the path most exposed to inflation under these conditions. Harman’s single-factor test, applied post hoc, is the weakest of the common CMV diagnostics (Richardson et al., 2009) and should not by itself be read as a clean bill of health for this path. Replication with individual, private administration—or with mixed self- and partner reports—would provide a cleaner estimate that the present design cannot.

Two measurement limitations specific to the empowerment instrument require explicit acknowledgment. Firstly, no formal cross-national measurement invariance was established between the Mexican normative IMWE sample and the Peruvian sample analyzed here. Score comparability across the two populations therefore rests on expert-panel face adaptation rather than on configural, metric, or scalar invariance evidence, and between-population comparisons should be made cautiously. Secondly, the Equality subscale fell marginally below the conventional α ≥ 0.70 benchmark (α = 0.699), which means that inferences touching the equality dimension are held to a weaker psychometric standard than the rest of the instrument. Full CFA and multigroup invariance testing against the Mexican normative data are recommended prerequisites for cumulative use of the IMWE in Peruvian research, and the present findings involving empowerment—including the threshold pattern in Section 3.7, which is anchored to the IMWE normative cut-point—should be read against these open psychometric questions.

Sixthly, the null effects of “disengagement” on satisfaction might reflect measurement limitations. The combined CSI-SF subscale (α = 0.89) amalgamates problem-focused and emotion-focused avoidance, perhaps inappropriate for culturally relevant forms of avoidance (e.g., religious fatalism or resignation, deference to male authority, community withdrawal). Culturally adapted instruments are required.

Seventhly, the present study did not test the pathways through which disengagement harms well-being. The significant motherhood moderation (β = −0.272, *p* = 0.021) raises the question of whether this effect operates via resource depletion, role conflict, or social isolation—mechanisms that diary studies or experimental designs could disentangle.

Eighthly, the binary coding of partnership status is itself a limitation. The unpartnered group combined women who had never married with those separated, divorced, or widowed, and the psychological realities of these subgroups differ substantially: a young woman who has never partnered is not in the same life situation as a woman two years into widowhood (Amato, 2010). The large partnership coefficient (β = 0.734) therefore probably mixes the gains of active partnering with the losses tied to dissolution and bereavement, and the present data cannot separate the two. A disaggregated analysis was not feasible given the cell sizes available here; future work should model marital status categories separately to clarify what the partnership variable is actually measuring.

Finally, intervention trials that achieve ‘threshold crossings’ of empowerment and RCT evidence comparing multicomponent vs. standard programmers are prioritized, along with qualitative studies that identify psychological, social and material factors that facilitate crossing. The strong effect of cohabitation/partnership on satisfaction suggests dyadic analyses of empowerment and engagement between partners. These adjustments would improve the internal and external validity of the findings substantially.

## 5. Conclusions

This study provides evidence that psychological empowerment is associated with life satisfaction among Peruvian women via coping engagement, consistent with a mediational mechanism—specifically, active, approach-oriented coping strategies including problem-focused and emotion-focused engagement. The indirect effect through coping engagement was significant (β = 0.134, 95% CI [0.065, 0.213]), while the direct effect was not, indicating full mediation. Exploratory threshold analyses suggest that only high empowerment levels (≥136) significantly predict life satisfaction, though this finding requires replication. Coping disengagement operates largely outside the empowerment–satisfaction pathway, although it becomes particularly harmful for mothers. Sociodemographic factors—particularly age, motherhood, and partnership status—introduce heterogeneity in how empowerment processes unfold.

The first aim of the study—clarifying whether coping engagement carries the empowerment signal into life satisfaction—is supported in substance, with the caveat that the cross-sectional design licenses a mechanistic reading rather than a causal one. The second aim—mapping how sociodemographic circumstances reshape that pathway—was partially met: age and motherhood emerged as meaningful conditioners; partnership and employment did not, though the power available to detect small interaction effects was limited. The third aim—probing a threshold above which empowerment begins to register in well-being—produced a pattern that is suggestive rather than settled, and that belongs in the hypothesis-generating column until replication with an independently derived cut-point. Taken together, the three aims are fulfilled in the sense that each yielded a defensible inferential outcome, but none of the findings should be carried forward as if a single study had closed the question.

The three aims of the study were fulfilled to varying degrees. The primary aim—establishing whether coping engagement mediates the empowerment–life satisfaction relationship—was supported, with the pattern statistically consistent with full mediation. The second aim—mapping how sociodemographic circumstances condition that pathway—was partially met: age and motherhood emerged as meaningful moderators, while partnership and employment did not, partly reflecting limited statistical power for small interaction effects. The third aim—probing a possible empowerment threshold—produced a suggestive but provisional pattern that belongs in the hypothesis-generating column pending independent replication. Collectively, the study advances understanding of how psychological resources translate into well-being in high-informality Latin American contexts, while underscoring that cross-sectional evidence licenses mechanistic interpretations rather than causal conclusions. Each finding should be treated as a step in a cumulative research program rather than a settled answer.

Exploratory threshold analysis—anchored to a normative cut-point drawn from the IMWE manual rather than an empirically optimized boundary—suggested that the empowerment–satisfaction association emerged mainly among women at or above that cut-point (≥136), while below it, the slope was essentially flat. A quadratic sensitivity check was non-significant, so a linear or null continuous relationship remains equally plausible; the threshold pattern is best read as hypothesis-generating rather than as an established discontinuity. If subsequent replications with independently derived cut-points confirm it, the practical implication would be that this pattern challenges assumptions that guide incremental interventions and suggests that programs must reach critical intensities—simultaneously addressing the seven dimensions of empowerment (courage, independence, equality, social satisfaction, security, participation, and external influences)—to achieve significant improvements in well-being. Marginal or single-component strategies may be insufficient, as they do not raise empowerment to the level at which engagement mechanisms are activated.

Moderation effects show complex contextual nuances. Age significantly moderated the engagement–satisfaction link (β = −0.239, *p* = 0.031): younger women (≤32 years) show stronger associations than older women. This points to generational differences in how engagement translates into well-being, possibly linked to expectations, opportunities, and role definitions. Motherhood accentuated the negative impact of disengagement on life satisfaction (β = −0.272, *p* = 0.021), with more severe consequences for mothers than for non-mothers. This result is consistent with Conservation of Resources theory: those who already operate with limited resources suffer particularly adverse effects when they resort to withdrawal strategies, because disengagement further depletes the resources available to meet concurrent demands.

Cohabitation or relationship status had a notable direct effect on life satisfaction (β = 0.734, *p* < 0.001): women in relationships report much higher levels of satisfaction than single, divorced, or widowed women. However, relationship status did not significantly moderate the pathways of engagement or disengagement, suggesting that although having a partner increases satisfaction—probably due to companionship, emotional support, and economic resources—it does not fundamentally change the functioning of empowerment and coping processes. Employment status did not show significant effects, either as a direct predictor of satisfaction or as a moderator of the empowerment–engagement relationship, perhaps because, in environments where informal work predominates, employment status has less psychological salience than in established formal labor markets.

The implications for policies and interventions are clear. Programs must simultaneously promote the development of empowerment and the creation of opportunities for engagement, understanding that internal resources only produce improvements when they are converted into active participation. Interventions must be sufficiently intense and multidimensional to push participants above critical thresholds, avoiding gradual approaches with limited scope. In addition, they must be adapted to the needs identified in the moderation analyses: for mothers, it is advisable to reduce disengagement while strengthening engagement; for younger women, to take advantage of the particularly strong link between engagement and satisfaction; and to consider marital status as a relevant determinant of well-being, complementing this with programs on relationship quality or social connection initiatives for single or divorced women.

The findings make three contributions. Theoretically, they specify the operative mechanism: coping engagement, not empowerment itself, drives life satisfaction gains, and this pathway functions only above a critical empowerment level—extending Hobfoll’s (2002) Conservation of Resources model to threshold dynamics under conditions of structural disadvantage. Methodologically, the non-significant IMM indices call for precision in how combined mediation-and-moderation designs are labeled. Practically, the results identify mothers and younger women as the subgroups for whom coping engagement most critically determines well-being. Three research priorities follow: randomized trials testing whether multicomponent programs can move participants above the critical empowerment threshold; longitudinal panels to establish temporal precedence; and qualitative work mapping the conditions that block coping engagement among informally employed mothers—the subgroup this study identifies as most exposed to the costs of insufficient empowerment.

## Figures and Tables

**Table 1:** Descriptive statistics and bivariate correlations.

Variable	M	SD	Range	1	2	3	4	5	6	7	8
1. Empowerment	3.77	0.54	2.29–5.00	—							
2. Engagement	3.68	0.79	1.00–5.00	0.26 **	—						
3. Disengagement	2.29	0.88	1.00–5.00	−0.15 *	−0.09	—					
4. Life satisfaction	4.82	1.37	1.00–7.00	0.19 **	0.44 **	−0.04	—				
5. Age ≤ 32 years	—	—	0–1	0.05	0.03	−0.03	−0.02	—			
6. Partnered	—	—	0–1	0.08	0.10	−0.03	0.61 **	0.01	—		
7. With children	—	—	0–1	0.09	0.03	−0.03	0.09	0.17 *	0.14 *	—	
8. Employed	—	—	0–1	0.09	0.03	−0.05	0.08	0.11	0.10	0.06	—

Note. *N* = 251. M = mean; SD = standard deviation. For continuous variables, means and standard deviations represent scale means (averaged across items) to facilitate interpretation: Empowerment = mean of 34 items on 1–5 scale (total score range 34–170, M = 128.34, SD = 18.45); engagement = mean of 8 items on 1–5 scale; disengagement = mean of 8 items on 1–5 scale; life satisfaction = mean of 5 items on 1–7 scale (total score range 5–35, M = 24.12, SD = 6.83). Actual ranges observed: Empowerment 2.29–5.00 (78–170 total score); life satisfaction 1.00–7.00 (5–35 total score). Dichotomous variables: 0 = no, 1 = yes. All participants were aged 18-44 years by design (continuous age: M = 32.6, SD = 7.4). Partnered: 64.9%; with children: 71.3%; employed: 58.2%. ** *p* < 0.01, * *p* < 0.05 (two-tailed).

**Table 2:** Construct reliability and convergent validity.

Construct	Number of Indicators	Cronbach’s α	Composite Reliability (ρc)	AVE
Empowerment	7	0.769	0.722	0.575
Engagement	8	0.804	0.797	0.814
Disengagement	8	0.798	0.832	0.713
Life satisfaction	5	0.852	0.894	0.630

Note. *N* = 251. AVE = average variance extracted. All values meet or exceed recommended thresholds (α ≥ 0.70, ρc ≥ 0.70, AVE ≥ 0.50; Hair et al., 2019).

**Table 3:** Variance inflation factors for structural model predictors of life satisfaction.

Predictor Construct	VIF	Tolerance	Assessment
Empowerment	1.15	0.870	Excellent
Engagement	1.42	0.704	Excellent
Disengagement	1.08	0.926	Excellent
Age (18–32 years)	1.06	0.943	Excellent
Partnered	1.73	0.578	Excellent
With children	1.11	0.901	Excellent
Employed	1.07	0.935	Excellent

Note. *N* = 251. VIF = variance inflation factor; tolerance = 1/VIF. Values represent collinearity assessment for predictor constructs in the structural model (inner model) predicting life satisfaction. All VIF values < 3.0 indicate absence of problematic multicollinearity (Hair et al., 2019). Assessment categories: VIF < 2.0 (tolerance > 0.50) = excellent, no multicollinearity concerns; VIF 2.0–3.0 (tolerance 0.33–0.50) = acceptable, minimal multicollinearity; VIF 3.0–5.0 (tolerance 0.20–0.33) = moderate multicollinearity, warrants caution; VIF > 5.0 (tolerance < 0.20) = critical multicollinearity, predictor should be removed or combined. Values were extracted from SmartPLS 4.0 inner model collinearity statistics. The highest VIF (Partnered = 1.73) remains well below critical thresholds, confirming that predictor constructs exhibit sufficient discriminant validity and do not compromise structural parameter estimates.

**Table 4:** Structural model fit indices.

Fit Index	Value	Threshold	Interpretation
SRMR	0.078	≤0.08	Good fit
NFI	0.942	≥0.90	Acceptable fit
d_ULS	1.215	≤2.0	Acceptable fit
d_G	0.312	≤0.5	Acceptable fit
Chi-square	185.826 (df = 142)	*p* > 0.05 (ns)	Acceptable *

Note. *N* = 251. SRMR = standardized root means square residual; NFI = normed fit index; d_ULS = unweighted least squares discrepancy; d_G = geodesic discrepancy. ns = non-significant. * Chi-square derived from exact fit test via bootstrapping; this statistic is sensitive to sample size and should be interpreted alongside other fit indices. Given acceptable SRMR and NFI values, the model is considered adequate (Hair et al., 2019).

**Table 5:** Direct effects: path coefficients, effect sizes, and variance explained.

Hypothesis/Path	β	SE	t	*p*	f^2^	95% CI	Decision
**Direct pathway hypotheses**							
H1: Empowerment → Engagement	0.264	0.066	4.036	<0.001	0.075	[0.135, 0.393]	Supported
H2: Engagement → Life satisfaction	0.508	0.134	3.782	<0.001	0.348	[0.245, 0.771]	Supported
H3: Empowerment → Disengagement	−0.149	0.084	1.785	0.074	0.023	[−0.314, 0.016]	Not supported
H4: Disengagement → Life satisfaction	−0.123	0.177	0.697	0.486	0.016	[−0.470, 0.224]	Not supported
H5: Empowerment → Life satisfaction	0.019	0.061	0.314	0.754	<0.001	[−0.100, 0.138]	Not supported
**Sociodemographic controls**							
Age (≤32 years) → Life satisfaction	−0.032	0.185	0.175	0.861	0.001	[−0.395, 0.331]	ns
Partnered → Life satisfaction	0.734	0.199	10.592	<0.001	0.587	[1.719, 2.499]	Significant
With children → Life satisfaction	−0.062	0.200	0.307	0.759	0.001	[−0.454, 0.330]	ns
Employed → Life satisfaction	0.058	0.223	0.261	0.794	0.001	[−0.379, 0.495]	ns

Note. *N* = 251. β = standardized path coefficient; SE = standard error derived from 5000 bootstrap samples; t = t-statistic; *p* = two-tailed significance; f^2^ = Cohen’s effect size (0.02 = small, 0.15 = medium, 0.35 = large); CI = confidence interval; ns = not significant. R^2^ for engagement = 0.070; R^2^ for disengagement = 0.022; R^2^ for life satisfaction = 0.285.

**Table 6:** Moderation effects: interaction terms and simple slopes analysis.

Hypothesis/Interaction	β	SE	t	*p*	f^2^	95% CI	Decision
**Age moderators**							
H6: Age × Engagement → Life satisfaction	−0.239	0.111	2.158	0.031	0.063	[−0.457, −0.021]	Supported
*Simple slope (Age ≤ 32)*	0.624	0.153	4.078	<0.001	—	[0.324, 0.924]	—
*Simple slope (Age > 32)*	0.385	0.163	2.362	0.018	—	[0.065, 0.705]	—
H7: Age × Disengagement → Life satisfaction	−0.023	0.103	0.222	0.824	0.001	[−0.225, 0.179]	Not supported
**Partnership moderators**							
H8: Partnered × Disengagement → Life satisfaction	−0.003	0.102	0.029	0.977	<0.001	[−0.203, 0.197]	Not supported
H9: Partnered × Engagement → Life satisfaction	−0.143	0.106	1.355	0.175	0.020	[−0.351, 0.065]	Not supported
**Motherhood moderators**							
H10: With children × Disengagement → Life satisfaction	−0.272	0.117	2.317	0.021	0.081	[−0.502, −0.042]	Supported
*Simple slope (Mothers)*	−0.395	0.138	2.862	0.004	—	[−0.666, −0.124]	—
*Simple slope (non-mothers)*	−0.123	0.130	0.946	0.342	—	[−0.378, 0.132]	—
H11: With children × Engagement → Life satisfaction	−0.093	0.099	0.941	0.347	0.010	[−0.287, 0.101]	Not supported
**Employment moderators**							
H12: Employed × Disengagement → Life satisfaction	0.190	0.156	1.220	0.223	0.041	[−0.116, 0.496]	Not supported
H13: Employed × Engagement → Life satisfaction	−0.004	0.115	0.034	0.973	<0.001	[−0.230, 0.222]	Not supported

Note. *N* = 251. β = standardized interaction coefficient; SE = standard error from 5000 bootstrap samples; f^2^ = Cohen’s effect size for interaction; CI = confidence interval. Simple slopes computed at ±1 SD for continuous moderators or at 0/1 for dichotomous moderators. Simple slopes displayed only for significant interactions.

## Data Availability

The fully anonymized dataset, codebook, and SmartPLS syntax supporting the findings of this study are publicly available in Figshare at https://doi.org/10.6084/m9.figshare.32227176, in accordance with the journal’s open-data policy.

## Supplementary Materials

The following supporting information can be downloaded at: https://www.mdpi.com/article/10.3390/ejihpe16060085/s1, Table S1: Continuous-age moderation sensitivity analysis: interaction terms and simple slopes with age as a z-standardized predictor.
