# Supplementary material for: Coping Engagement as the Pathway from Psychological Empowerment to Life Satisfaction: A Mediation and Moderation Independent Analyses Among Women in Northern Peru
Source: Eur J Investig Health Psychol Educ. 2026 Jun 19;16(6):85. doi: 10.3390/ejihpe16060085 (PMC13298023; doi:10.3390/ejihpe16060085)
Supplement: Supplementary file 1 [file ejihpe-16-00085-s001.zip › ejihpe-4248266-supplementary.pdf]

Supplementary Table S1. Continuous-age moderation sensitivity analysis: interaction terms and simple slopes with age as a z-standardized predictor.

Hypothesis/Interaction	β	SE	t	p	f^2	95% CI	Decision
Age × Engagement → Life satisfaction	−.183	.090	2.033	.043	.051	[−.359, −.007]	Supported
Simple slope (Age = −1 SD, ≈25 yrs)	*.697*	*.142*	*4.908*	*< .001*	—	*[.419, .975]*	—
Simple slope (Age = mean, ≈33 yrs)	*.514*	*.131*	*3.924*	*< .001*	—	*[.257, .771]*	—
Simple slope (Age = +1 SD, ≈40 yrs)	*.331*	*.145*	*2.283*	*.023*	—	*[.047, .615]*	—
Age × Disengagement → Life satisfaction	−.021	.088	0.239	.812	.001	[−.193, .151]	Not supported

Note. N = 251. Age was z-standardized prior to interaction-term computation (M = 32.6 years, SD = 7.4, range 18–44). β = standardized interaction coefficient; SE = bootstrap standard error from 5000 resamples; CI = 95% percentile bootstrap confidence interval. Simple slopes for the engagement–life satisfaction relationship were probed at −1 SD, mean, and +1 SD of the continuous-age moderator. Conclusions are equivalent to those obtained with the dichotomized specification reported in Table 6: the Age × Engagement interaction is significant in the same direction (categorical β = −.239, p = .031; continuous β = −.183, p = .043), and the Age × Disengagement interaction remains non-significant in both specifications. Estimates from the continuous model are slightly attenuated relative to the dichotomized model, consistent with expected variance recovery (MacCallum et al., 2002). The partial measurement invariance caveat for engagement across age subgroups noted in Section 3.2 applies equally to this continuous sensitivity analysis; replication with an age-balanced design and full configural, metric, and scalar invariance is recommended before drawing strong conclusions about age as a moderator of the engagement–life satisfaction pathway.
